# Emergency general surgery in *BJS Open*—papers of note in 2022

**DOI:** 10.1093/bjsopen/zrad004

**Published:** 2023-02-13

Emergency surgery is rising in profile around the world, with professionalization as a subspecialty, and is the focus of many learned societies. There is still much progress to be made in research in this field, and we should welcome all those who strive to further knowledge and improve care here. I was pleased to be asked to select my favourite emergency surgery papers from *BJS Open* in 2022. Across the six issues last year, there have been some interesting and debate-stimulating papers published, using different designs and approaches to clinically important questions. After some deliberation, I have settled on the three below as ‘must reads’.

My first recommendation is the scoping review by Javanmard-Emamghissi *et al.*, published in April 2022^1^. As futility is a key concept for the emergency general surgeon, one might hope that we could all agree on the definition for it. This scoping review identified just three papers on the topic, all of which focused on the idea of ‘quantitative futility’, that is death within two days of treatment. Readers may find this jarring, as it highlights a blind spot around ‘qualitative’ futility, despite this being an often-used reason to avoid intervention in practice. Perhaps this isn’t a surprise given that the emergency literature often fails to include patient-reported outcomes, and that death is an easy metric to measure. The article should encourage a wider exploration of the meaning and impact of futility in the emergency surgical setting.

Quantitative analysis is a commonly used approach to explain study findings, providing *P* values and confidence intervals. However, it doesn’t always provide the context or nuance to understand what is happening behind the numbers. This leads to the second selection, the paper ‘*Recruiting to surgical trials in the emergency setting: using a mixed methods study to understand clinician and patient perspectives*’ by Twiddy *et al.*^2^.This reports surveys and interviews from patients and clinicians involved in the LACES trial (a UK-based feasibility trial comparing laparoscopic with open emergency colorectal surgery). It provides useful insights into challenges to recruitment and equipoise for emergency trials, including the role of treating clinician attitudes, and the importance of trainee surgeons in effective recruitment to trials. It is likely this will become a key reference paper for those developing grant applications and research protocols for emergency surgery studies.

Finally, management of foreign body ingestion is often led by surgeons. In June, the Paediatric Surgery Trainee Research Network published an observational cohort study on magnet and button battery ingestion^3^. Ingestion of items such as these has increased in recent years, and presents a challenge for the responsible clinician, balancing the need for intervention *versus* unnecessary delay. This study reports on 263 events of magnet or button battery ingestion. Key findings from this manuscript include an almost 50 per cent surgical or endoscopic intervention rate for magnet retrieval, and button batteries causing morbidity or life threatening events in 14 per cent and 2 per cent of patients respectively. This paper was selected as it demonstrates real world management and outcomes in a condition that can still cause angst and uncertainty for surgeons in desperate need of a solid evidence base to support best care.

Honourable mentions go to Herrod *et al.* for their meta-analysis of antibiotic therapy for appendicitis^4^, Stieler *et al.* for an interesting paper on somatic syndromes in acute presentations^5^, and Soreide *et al.* for a surgical education piece on learning curves for laparoscopic appendicectomy^6^. We look forward to reading future high-quality emergency surgery papers in *BJS Open*.

## References

[1] Javanmard-Emamghissi H , Lockwood S, Hare S, Lund JN, Tierney GM, Moug SJ. The false dichotomy of surgical futility in the emergency laparotomy setting: scoping review. BJS Open 2022;6:zrac023 10.1093/bjsopen/zrac023 PMC8988868 35389427

[2] Twiddy M , Birtwistle J, Edmondson A, Croft J, Gordon K, Meads D et al Recruiting to surgical trials in the emergency setting: using a mixed methods study to understand clinician and patient perspectives. BJS Open 2022;6:zrac137 10.1093/bjsopen/zrac137 PMC9683391 36417312

[3] Paediatric Surgery Trainee Research Network . Magnet and button battery ingestion in children: multicentre observational study of management and outcomes. BJS Open 2022;6:zrac056 10.1093/bjsopen/zrac056 PMC9164827 35657136

[4] Herrod PJJ , Kwok AT, Lobo DN. Randomized clinical trials comparing antibiotic therapy with appendicectomy for uncomplicated acute appendicitis: meta-analysis. BJS Open 2022;6:zrac100 10.1093/bjsopen/zrac100 PMC9379374 35971796

[5] Stieler M , Pockney P, Campbell C, Thirugnanasundralingam V, Gan L, Spittal MJ et al Somatic symptom severity association with healthcare utilization and costs in surgical inpatients with an episode of abdominal pain. BJS Open 2022;6:zrac046 10.1093/bjsopen/zrac046 PMC9260183 35796068

[6] Søreide K , Skjold-Ødegaard B. A multilevel, step-based model to evaluate progress in procedure efficiency for laparoscopic appendicectomy in surgical training: structured evaluation using ‘ebb-and-flow’ and ‘string-of-pearls’ concepts. BJS Open 2022;6:zrac071 10.1093/bjsopen/zrac071 PMC9176202 35674702

